# Deciphering the Black Box of Midlife Aging: Unravelling Functional Changes in the Aging Process

**DOI:** 10.1093/geroni/igaf122.959

**Published:** 2025-12-31

**Authors:** Maayan Agmon

## Abstract

Midlife is a pivotal yet understudied phase of the aging process, offering a unique opportunity to identify early markers of functional decline and implement interventions that promote healthy aging. This symposium presents multidisciplinary research examining the influence of personal and environmental factors as key determinants of midlife function and aging trajectories. Professor Agmon will provide an overview of the spectrum of personal and environmental factors studied in her lab, emphasizing newly developed behavioral markers that can be used to quantify function in midlife. Roy Tzemah-Shahar and Merav Asher will each present studies exploring the effects of gait characteristics, sensory responsiveness, and emotion regulation on midlife function, comparing different aging markers (biological age vs. physical capacity). Khalil Iktilat will present an examination of the impact of environmental factors, such as exposure to violence, on health disparities, manifested as accelerated biological aging in midlife. Finally, we will present the Midlife Aging and Performance Study (MAPS) which introduces a novel functional assessment battery linked to traditional biological age evaluation. Presented studies highlight the importance of integrating physiological, psychological, and behavioral markers to advance our understanding of midlife function and its long-term health implications. The findings emphasize the need for deeper scientific focus on midlife and targeted preventive interventions ensuring tailored strategies that enhance active aging and reduce disparities in diverse populations.

